# Abnormal Antennal Olfactory Sensilla Phenotypes Involved in Olfactory Deficit in *Bactrocera correcta* (Diptera: Tephritidae)

**DOI:** 10.3390/insects13060535

**Published:** 2022-06-10

**Authors:** Kai-Fei Guo, Xiao-Mei Peng, Jie-Yu Tu, Chan Jin, Wan-Rong Zhang, Xi-Zhu Chen, Yong-Jun Liu, Hong-Guang Zha, Wei Shi, Jun Cao

**Affiliations:** 1 Yunnan Key Laboratory of Plant Reproductive Adaptation and Evolutionary Ecology and Centre for Invasion Biology, Institute of Biodiversity, School of Ecology and Environmental Science, Yunnan University, Kunming 650504, China; gkf@yxnu.edu.cn (K.-F.G.); pxm17863997736@163.com (X.-M.P.); luckytututujieyu@163.com (J.-Y.T.); chanooos@163.com (C.J.); zhangwr91@163.com (W.-R.Z.); xzchen334@163.com (X.-Z.C.); shiwei@ynu.edu.cn (W.S.); 2 School of Chemistry, Biology and Environment, Yuxi Normal University, Yuxi 653100, China; 3 School of Chemical Science and Technology, Yunnan University, Kunming 650504, China; yjliu@ynu.edu.cn; 4 College of Life and Environment Sciences, Huangshan University, Huangshan 245041, China; zhahg@tech.hsu.edu.cn

**Keywords:** abnormal olfactory sensilla phenotypes, bulges, nanopores, olfactory deficit, *Bactrocera correcta*

## Abstract

**Simple Summary:**

Tephritidae fruit flies sense odorants mainly through antennal olfactory sensilla with nanopores. Therefore, theoretically, the development of nanopore-targeted pest control technologies is an important direction in the future. Here, we report naturally occurring abnormal antennal trichoid and basiconic olfactory sensilla phenotypes consisting of abnormal bulges and reduced nanopore numbers in a long-term laboratory rearing colony of the guava fruit fly *Bactrocera correcta*, and further find that the reduction of nanopore numbers in these sensilla led to an olfactory deficit. Our findings provide a basis for developing nanopore-targeted pest control technologies in the future.

**Abstract:**

The guava fruit fly, *Bactrocera correcta*, is one of the most destructive pests in the genus *Bactrocera* and detects environmental odorants mainly through antennal olfactory sensilla phenotypes with nanopores. However, it is unclear whether there are naturally occurring abnormal antennal olfactory sensilla phenotypes that affect olfaction. Here, we found that there were abnormal bulges besides nanopores on the surface of trichoid and basiconic olfactory sensilla in the antennal flagellum of long-term laboratory rearing colony (LTC), and that nanopore number in these olfactory sensilla was also remarkably reduced. Notably, the electroantennogram (EAG) responses of LTC insects to methyl eugenol or β-caryophyllene were inhibited, and their behavioral responses elicited by the same odorants were also impaired. These results revealed naturally occurring abnormal antennal olfactory sensilla phenotypes which were involved in olfactory deficit in *B. correcta,* providing a platform to further study nanopore-targeted pest control technologies in the future.

## 1. Introduction

The guava fruit fly, *Bactrocera correcta* (Bezzi) (Diptera: Tephritidae), is one of the most destructive pests in the genus *Bactrocera* [1,2], which is known to infest over 70 species of fruits and vegetables in 35 plant families [3,4,5]. The pest occurs throughout South and Southeast Asia including Nepal, Pakistan, Sri Lanka, India and Thailand [1,3,6], and is now invasively spreading to Burma, Vietnam and China [1,7]. Thus, it is considered to be highly invasive and listed as a quarantine pest species by countries [7].

It is well-known that normal insect olfactory sensilla phenotypes have numerous nanopores distributing on their surfaces [8,9]. Insects such as Tephritidae fruit flies sense volatile odorants originating from food sources and oviposition sites through nanopores in the cuticle wall of the olfactory sensilla [9,10,11,12,13]. Odorant molecules diffuse into the lumen of cuticular sensilla through nanopores and then bind to olfactory receptors expressed on olfactory sensory neurons [10,14]. Olfactory sensilla with numerous nanopores are vital for insect olfactory perception. Therefore, theoretically, regulation of olfactory sensilla phenotypes is an important potential direction for the development of pest control technologies in the future. However, discovering naturally occurring abnormal phenotypes in Tephritidae fruit flies is required for the study.

Existing studies from Tephritidae fruit flies focus on ultrastructure and morphology of antennal sensilla and olfactory receptors [13,15,16,17,18,19]. Although a 1994 report of the oriental fruit fly *Bactrocera dorsalis* (described as “*Dacus dorsalis*”) simply described an abnormal antennal trichoid olfactory sensilla phenotype consisting of bulges (described as “nobules”, probably “nodules”) and unclear nanopores, no explanation was provided [15]. Furthermore, whether and how these structural changes affected olfaction function were unclear [15].

In the present study, using advanced high-resolution field emission scanning electron microscopy, we reported abnormal antennal olfactory sensilla phenotypes and their features in a long-term laboratory rearing colony (LTC) of the guava fruit fly. We further examined whether and how the abnormal antennal olfactory sensilla phenotypes affected electroantennogram (EAG) responses and olfactory behavior.

## 2. Materials and Methods

### 2.1. Insects

*Bactrocera correcta* larvae were originally collected from fallen mango fruits in Yuanjiang County, Yunnan Province, China. Emerged adult flies were reared in cages under laboratory conditions with 25 ± 1 °C, 50–70% relative humidity and a L12:D12 photoperiod, fed with a mixture of sugar and yeast (3:1) and supplied water using soaked cotton wool. Guava fruits were provided for female oviposition and larval development. The long-term laboratory rearing colony (LTC) was the 20th to 24th generations from the wild collections. The short-term laboratory rearing colony was the second to fourth generations from the wild collections, and referred to as a wild colony (WC). Before the experiments, whether the main structures (e.g., wings and antennae) on the head, thorax and abdomen of the adult guava fruit fly were intact was grossly examined using a SZX16 stereoscopic microscope (Olympus, Tokyo, Japan). To examine whether abnormal antennal olfactory sensilla phenotypes affected olfactory behavior in a Y-tube olfactometer assay, specific and potent insect lures were needed. So far, only male lures of *B. correcta* have been found [20,21]. Thus, the male flies during 10–15 days after eclosion were used.

### 2.2. Chemicals

All chemicals were purchased from Sigma-Aldrich Chemicals (St. Louis, MO, USA). Pure preparation of methyl eugenol (≥99.8% purity) and β-caryophyllene (≥98% purity) were respectively diluted with paraffin oil to 1 μg/μL for electrophysiological and behavioral experiments.

### 2.3. Scanning Electron Microscopy

To eliminate nanoparticle dirt covering the surface of individual sensillum, the sample preparation protocol was optimized based on previous methods [13,22]. The isolated male antenna was individually placed in a solution of hexane and sonicated three times for 5 min each to remove surface wax and other dirty particles. The antenna was fixed in 2% glutaraldehyde for 2 h and then washed three times for 5 min each in a 0.1 M phosphate buffer. An antenna was dehydrated in 70, 80, 90 and 100% ethanol for 5 min each. Afterward, a sample was air dried for 5 min at room temperature and mounted on a specimen stub with sticky tapes. The sample was coated with gold film using a sputtering device (Hitachi E-1010, Tokyo, Japan) for 5 min. An advanced low-voltage field emission scanning electron microscope (FESEM, Nova NanoSEM 450, 10 kV, FEI Company, Brno, Czech) was used to obtain high-resolution imaging of an antenna. Sixteen insects from WC or LTC were respectively used to observe the morphological difference of right antennae between WC and LTC groups. Among them, six antennae from each group were used to further examine the differences in number and ultrastructure of olfactory sensilla between WC and LTC groups. The external and medial surfaces of the antennal flagellum were selected as sampling areas of olfactory sensilla. The flagellum length and width, sensillum types and the length and middle width and nanopore diameter of each type of olfactory sensillum were obtained. The number of each type of olfactory sensillum in the flagellum, and the number of nanopores on the visible surface area of each type of olfactory sensillum were calculated.

### 2.4. Electrophysiological Recordings

A custom electroantennogram (EAG) system was used to examine the responses of antennal sensory neurons to odorants [23]. Each antenna preparation with the tip cut open was mounted between the glass reference electrode and the glass recording electrode containing insect Ringer’s solution [24]. A purified and humidified air flow (speed 15 mL/s) was constantly blown over the antenna via a glass tube placed approximately 1 cm from the antenna. The tip of a Pasteur pipette containing a piece of filter paper (0.5 cm × 2 cm, Whatman qualitative, grade #1) loaded with 1 μL of liquid sample was inserted into a small hole in the glass tube. Odor stimulus was administered by injecting a puff of purified air (1 s at 5 mL/s airflow) at intervals of 30 s through the pipette using the stimulus delivery controller. One antenna from each individual was tested in turn by paraffin oil and odorant stimulus. Signals were amplified by a custom-made high sensitivity amplifier (Syntech, Buchenbach, Germany) and output into a HP34465A digital multimeter (Keysight, Penang, Malaysia) controlled by BenchVue software (Keysight, Penang, Malaysia). The resulting field potential EAG amplitude was computed as the difference between the EAG value of paraffin oil control and the EAG value of a stimulant. Since methyl eugenol and β-caryophyllene had been demonstrated to be specific and potent lures for males of *B. correcta* [20,21], these two odorants were chosen in electrophysiological and behavioral experiments. Fifteen insects from WC or LTC were respectively used for EAG experiments with each odorant.

### 2.5. Behavioral Bioassay

The behavioral responses to the two odorants were performed using a glass Y-tube olfactometer (internal diameter 2 cm, main stem 13 cm long, two arms each 13 cm long) previously described [25,26]. A piece of filter paper (0.5 cm × 2 cm) loaded with 5 μL of an odorant was placed at the end of one arm of the Y-tube. Another piece of filter paper of the same size, loaded with 5 μL of paraffin oil solvent, was placed to the end of the control arm. The speed of the purified and humidified inflow was 100 mL/min. To avoid bias, placement of the stimulant and the control was alternated between two arms for each run, and six replications were made for each odorant. In each replication, 20 healthy adults, which were starved for 12 h, were released at the main stem of the Y-tube and their distribution between two arms was recorded after 3 min. To avoid cross-contamination, a clean Y-tube was used for each odorant. The response rate was characterized as follows: total response rate = (T + C)/SUM [27] and right response rate = T/SUM, where T represents the number of flies in the treatment tube, C indicates the number of flies in the control tube and SUM is the number of flies tested.

### 2.6. Statistical Analysis

All data were presented as means ± standard error of the mean (S.E.M.). A threshold level of statistical significance was set at *p* < 0.05. An independent sample *t*-test was used to analyze the difference in antennal flagellum size, sensillum size, sensillum number, pore number, total response rate, right response rate and EAG amplitude (mv) to one odor between WC and LTC groups. One-way analysis of variance (*ANOVA*) followed by a Fisher test was used to analyze the difference in morphological characteristics of the antennal olfactory sensilla within LTC or WC groups.

## 3. Results

### 3.1. Antennal Sensilla of LTC and WC

**Figure 1:**
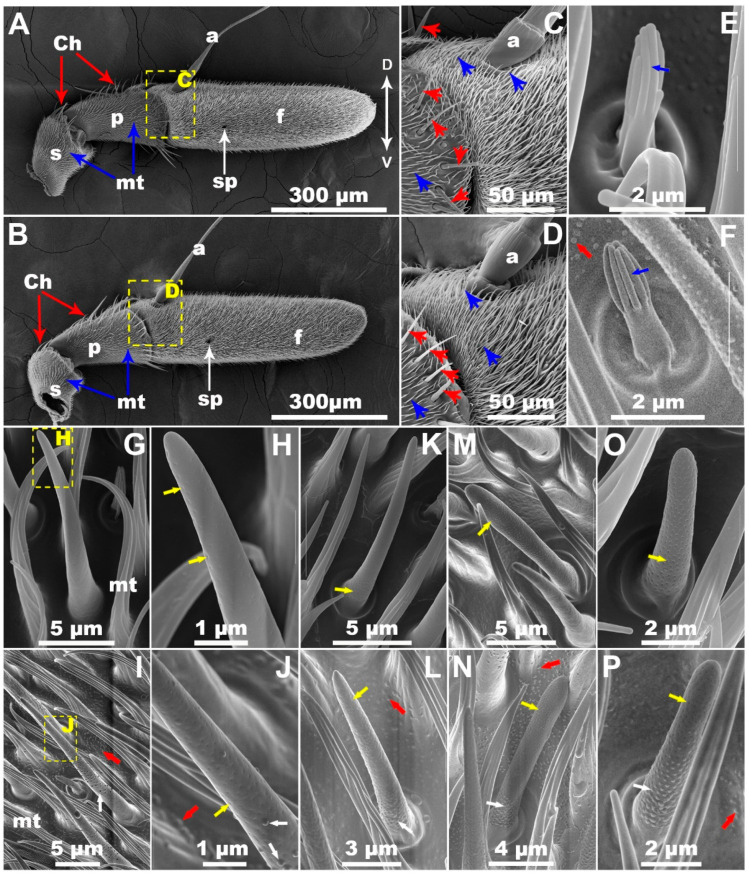
Scanning electron microscopy of the right antenna of males of *Bactrocera correcta*. (**A**) Lateral view of a WC antenna, and (**B**) that of a LTC antenna, showing three segments: scape (s), pedicel (p), flagellum (f), chaetic sensilla (Ch, red long arrow), microtrichia (mt, blue long arrow) and sensory pit (sp, white long arrow). (**C**) A magnified image of a part (yellow dotted box) of the WC antenna and (**D**) that of the LTC antenna, showing arista (a), chaetic sensilla (Ch, red arrowhead) and microtrichia (mt, blue arrowhead). (**E**) A representative image of WC grooved sensilla, showing smooth surface with longitudinal slits (blue arrow). (**F**) A representative image of LTC grooved sensilla, showing smooth surface with longitudinal slits (blue arrow). Bulges (red arrow) appeared on the antennal surface. (**G**,**H**) Representative images of WC trichoid Ⅰ sensilla, showing smooth surface with nanopores (yellow arrow). (**I**,**J**) Representative images of LTC trichoid Ⅰ sensilla, showing rough surface with bulges (white arrow) and nanopores (yellow arrow). Similar bulges (red arrow) also appeared on the antennal surface. (**K**) A representative image of WC trichoid Ⅱ sensilla, and (**L**) that of LTC trichoid Ⅱ sensilla. (**M**) A representative image of WC basiconic Ⅰ sensilla, and (**N**) that of LTC basiconic Ⅰ sensilla. (**O**) A representative image of WC basiconic Ⅱ, and (**P**) that of LTC basiconic Ⅱ sensilla. D, dorsal side; V, ventral side.

**Figure 2:**
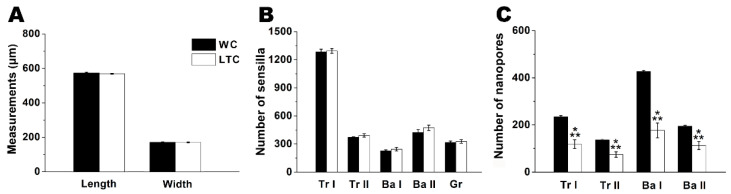
Comparations of flagellum size, sensilla number and nanopore number between WC and LTC groups. (**A**) The size of the antennal flagellum in WC and LTC groups. See Table S1 for detailed data. (**B**) The number of five morphological types of antennal olfactory sensilla in the WC and LTC groups. See Table S2 for detailed data. (**C**) Nanopore number of four morphological types of olfactory sensilla in the WC and LTC groups. See Table S3 for detailed data. *** *p* < 0.001.

Each antenna from the LTC and WC groups of *B*. *correcta* consisted of three segments (Figure 1A,B): scape, pedicel and an elongated flagellum. The flagellum had a long arista arising from the dorsoproximate region and a sensory pit located on the externo-lateral surface. The scape and the pedicel had dense microtrichia. There were rows of chaetic sensilla at the distal margin of these two segments and on the evagination of the pedicel at the flagellum. The flagellum was covered by dense, curved microtrichia (Figure 1A–D). There was no significant difference in the flagellum size (length: t = 1.156, df = 30, *p* = 0.256; width: t = 0.221, df = 30, *p* = 0.826, n = 16 antennae for each group, Figure 2A) between the LTC and WC groups. Our observation showed that LTC and WC had similar antennal morphology.

Based on FESEM images of olfactory sensilla (Figure 1E–P) and morphological data (Table 1), five morphological types of olfactory sensilla, grooved (Gr), trichoid Ⅰ (Tr Ⅰ), trichoid Ⅱ (Tr Ⅱ), basiconic Ⅰ (Ba Ⅰ) and basiconic Ⅱ (Ba Ⅱ) sensilla, were identified. These five types of olfactory sensilla were distributed on the flagellum of the LTC and WC groups. Moreover, as shown in Table 1, there was no significant difference in the size of grooved, trichoid Ⅰ, trichoid Ⅱ, basiconic Ⅰ or basiconic Ⅱ sensilla between the LTC and WC groups (Gr: length, t = 0.382, df = 38, *p* = 0.704; middle width, t = 0.061, df = 38, *p* = 0.952. Tr Ⅰ: length, t = 1.032, df = 38, *p* = 0.308; middle width, t = 0, df = 38, *p* = 1. Tr Ⅱ: length, t = 0.139, df = 38, *p* = 0.890; middle width, t = 0.206, df = 38, *p* = 0.838. Ba Ⅰ: length, t = 0.286, df = 38, *p* = 0.776; middle width, t = 0.236, df = 38, *p* = 0.815. Ba Ⅱ: length, t = 0.678, df = 38, *p* = 0.502; middle width, t = 0.165, df = 38, *p* = 0.870. n = 20 for each type of sensilla). There was no significant difference in the number of grooved, trichoid type (Ⅰ, Ⅱ), basiconic (Ⅰ, Ⅱ) olfactory sensilla between the LTC and WC groups (Gr: t = 0.365, df = 10, *p* = 0.722; Tr Ⅰ: t = 0.257, df = 10, *p* = 0.802; Tr Ⅱ: t = 1.023, df = 10, *p* = 0.330; Ba Ⅰ: t = 0.779, df = 10, *p* = 0.454; Ba Ⅱ: t = 1.175, df = 10, *p* = 0.267; n = 6 antennae for each group, Figure 2B). These results suggested that the LTC and WC groups had similar characteristics in these aspects.

Nanopores were found on the surface of trichoid (Ⅰ, Ⅱ) and basiconic (Ⅰ, Ⅱ) sensilla from the WC group (Figure 1G,H,K,M,O). However, a number of bulges besides nanopores appeared on the surface of these types of sensilla from the LTC group (Figure 1I,J,L,N,P). Similar bulges were also found on the antennal surface in LTC (Figure 1F,I,J,L,N,P). In addition, different collapse-like bulges appeared on the surface of basiconic sensilla (Figure S1). Moreover, there was significant difference in nanopore number of trichoid (Ⅰ, Ⅱ) and basiconic sensilla (Ⅰ, Ⅱ) between the LTC and WC groups (Tr Ⅰ: t = 6.103, df = 38, *p* < 0.001; Tr Ⅱ: t = 5.212, df = 38, *p* < 0.001; Ba Ⅰ: t = 7.788, df = 38, *p* < 0.001; Ba Ⅱ: t = 4.703, df = 38, *p* < 0.001, n = 20 for each type of sensilla, Figure 2C). These results revealed that LTC had abnormal antennal olfactory sensilla phenotypes consisting of bulges and reduced nanopore number.

### 3.2. The Abnormal Antennal Olfactory Sensilla Phenotypes Inhibited Antenna Function

**Figure 3:**
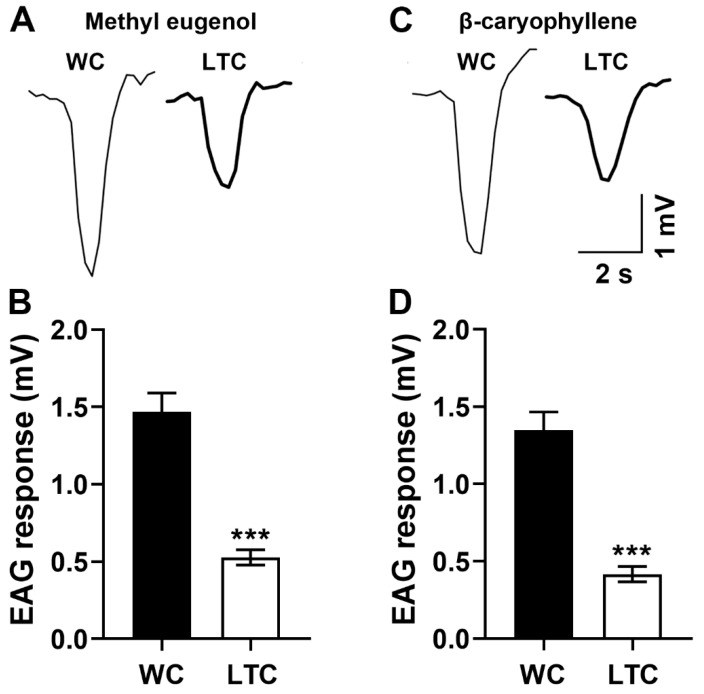
Inhibited EAG responses to methyl eugenol or β-caryophyllene in LTC. (**A**) Representative EAG responses of WC and LTC groups to methyl eugenol. (**B**) Reduced EAG amplitude to methyl eugenol in LTC. (**C**) Representative EAG responses of the WC and LTC groups to β-caryophyllene. (**D**) Reduced EAG amplitude to β-caryophyllene in LTC. See Table S4 for detailed data. *** *p* < 0.001.

We examined whether and how the abnormal antennal olfactory sensilla phenotypes affected antennal electrophysiological responses. There was significant difference in EAG amplitude between the LTC and WC groups when the antennal flagellum was respectively exposed to methyl eugenol (t = 6.353, df = 28, *p* < 0.001, n = 15 insects for each group, Figure 3A,B) or β-caryophyllene (t = 7.335, df = 28, *p* < 0.001, n = 15 insects for each group, Figure 3C,D). These results suggested that the abnormal antennal olfactory sensilla phenotypes inhibited the antenna function of the guava fruit fly.

### 3.3. The Abnormal Antennal Olfactory Sensilla Phenotypes Impaired Olfactory Behavior

**Figure 4:**
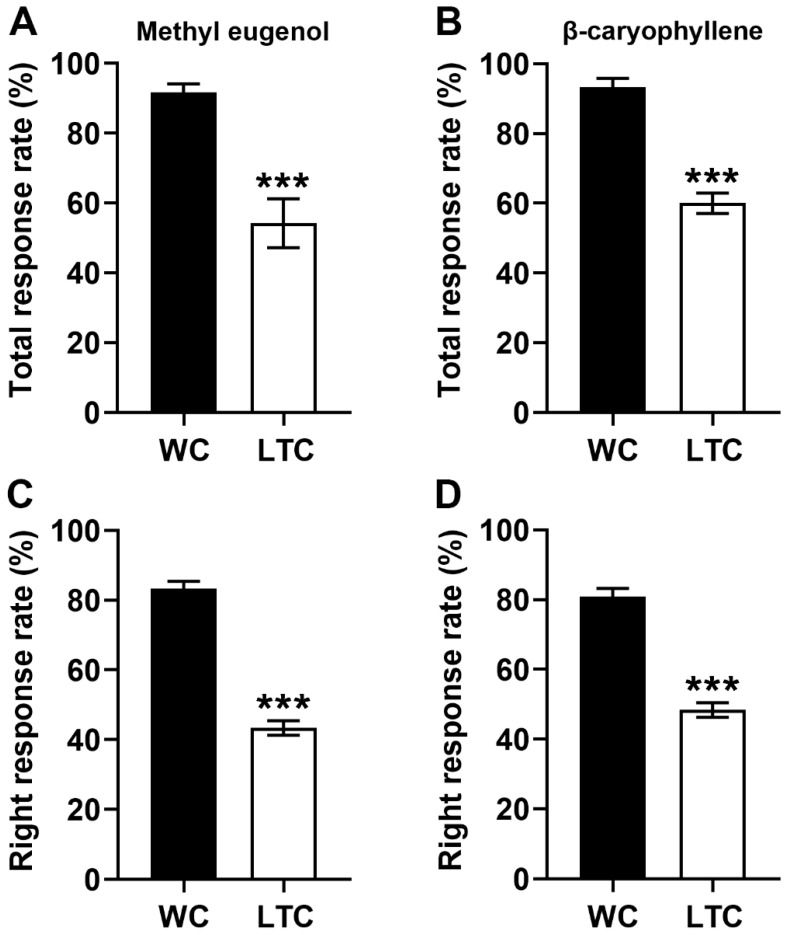
Impaired behavioral responses to methyl eugenol or β-caryophyllene in LTC. (**A**) Reduced total response rate to methyl eugenol in the LTC group. (**B**) Reduced total response rate to β-caryophyllene in the LTC group. (**C**) Reduced right response rate to methyl eugenol in the LTC group. (**D**) Reduced right response rate to methyl eugenol in the LTC group. See Table S5 for detailed data. *** *p* < 0.001.

To further determine whether and how the abnormal olfactory sensilla phenotypes affected olfactory behavior, we examined whether methyl eugenol or β-caryophyllene at a concentration of 1 µg/µL could still attract males of *B. correcta* using a Y-tube olfactometer. Compared with the WC group, the LTC group had a lower response rate to methyl eugenol (t = 7.758, df = 10, *p* < 0.001, n = 6 replications for each group, Figure 4A) or β-caryophyllene (t = 8.771, df = 10, *p* < 0.001, n = 6 replications for each group, Figure 4B). Furthermore, the LTC group had a lower right response rate to methyl eugenol (t = 13.416, df = 10, *p* < 0.001, n = 6 replications for each group, Figure 4C) or β-caryophyllene (t = 10.207, df = 10, *p* < 0.001, n = 6 replications for each group, Figure 4D). These results suggested that the abnormal antennal olfactory sensilla phenotypes led to an olfactory deficit of the guava fruit fly.

## 4. Discussion

The major findings of our study are that there are naturally occurring abnormal antennal trichoid (Ⅰ, Ⅱ) and basiconic (Ⅰ, Ⅱ) olfactory sensilla phenotypes with abnormal bulges and reduced nanopore number in a long-term laboratory rearing colony of *B. correcta.* Furthermore, the abnormal olfactory sensilla phenotypes are involved in decreased EAG responses and impaired olfaction.

In the present study, FESEM images in LTC of *B. correcta* clearly showed that there were bulges in the wall of the cuticular hair of antennal trichoid Ⅰ, trichoid Ⅱ, basiconic Ⅰ and basiconic Ⅱ olfactory sensilla. The present results in trichoid Ⅰ sensilla are similar to those from a 1994 report on *B. dorsalis* [15]. However, our results were inconsistent with those from numerous studies [8,9,13]. Furthermore, we have found decreased nanopore numbers on these olfactory sensilla in LTC. This is the first report on naturally occurring abnormal antennal olfactory sensilla phenotypes with bulges and reduced nanopore numbers.

It is well-known that the surface of insect olfactory sensilla has only nanopores [8,9,10,14]. The presence of cuticular bulges on the surface of olfactory sensilla in LTC of *B. correcta* requires explanations. Due to a lack of development evidence during the pupal stage, it is difficult for us to clearly explain how the bulges form and what the relationship between bulges and nanopores is. Four possibilities may be advanced. The first possibility is that concave nanopores may have everted from inward to outward during the pupal stage and the bulges formed. The second possibility is that the auxiliary cell of the sensillum may secrete a substance through nanopores, and then the bulges form. The third possibility is that the formation of bulges and nanopores may be respectively independent. The fourth possibility is that bulge formation may be related to nanopore formation. It is believed that insect cuticles including walls of insect olfactory sensilla are organized in a layered structure consisting of an envelope, epicuticle and chitin-rich procuticle, which are secreted by epidermal cells in a stepwise manner during the pupal stage [12,28,29,30,31]. A study from *Drosophila melanogaster* (Meigen) (Diptera: Drosophilidae) further shows that cuticular nanopores originate from a curved thin film that is formed in the outermost envelope of the cuticle and secreted from specialized protrusions in the plasma membrane of epidermal cells (often described as trichogen cells) [12]. Here, in our study, different collapse-like cuticular bulges appear on the sensilla surface during the adult stage (Figure S1). The collapse-like feature seems to be similar to the curvature of the envelope fragments during the pupal stage of *D. melanogaster* [12]. The cuticular bulges may be first secreted by trichogen cells during the pupal stage and then nanopores originate from the collapse of the bulges. The collapse-like processes of some bulges may be interrupted during the pupal stage for some unknown reasons. Different collapse-like bulges and pits (nanopores) will coexist on the olfactory sensilla. Anyway, the formation of the two structures and their relationship remain elusive. Further studies will be needed to verify which is the case using transmission electron microscopy (TEM). Interestingly, similar bulges also appear on the surface of antennal cuticle in LTC. To our knowledge, no studies report them. Due to no information being available, we cannot explain how the bulges on the antennal surface are formed by epidermal cells, and whether the formation of the antennal bulges and the sensilla bulges is different. The formation of the structure needs be examined using TEM in the future.

*Osiris* gene families found in a number of insect genomes are highly conserved among insects [32,33,34]. Furthermore, *Osiris* genes are expressed mainly in cuticle-secreting epidermal cells at cuticle-secretion stages [12,34]. Previous studies have shown that the *Osiris* 23 gene, also known as gore-tex, may drive the formation of the porous structures of insects [12,35]. The function of the gore-tex gene is required to introduce curvature into the newly formed envelope fragments prior to nanopore formation [12]. Gore-tex protein mainly localizing to the endosomes of trichogen cells of olfactory sensilla may help to pattern the endocytosis of the olfactory trichogen cell membrane and the curvature of the envelope fragments [12]. Thus, we speculate that the mutation of *Osiris* gene families in LTC of *B. correcta* may be vital for the occurrence of abnormal olfactory sensillum phenotypes and may affect the endocytosis of the olfactory trichogen cell membrane. It will be required to examine the mutation in *Osiris* gene families of LTC using sequencing technologies and to study related gene functions using the CRISPR/Cas9 system in the future.

A recent study of *D. melanogaster* has demonstrated that the sensory function of olfactory sensilla in maxillary palps is lost without nanopores [12], suggesting that external odorant molecules diffuse into the lumen of olfactory sensilla through nanopores. Here, in our study, reduction of nanopore numbers in LTC of *B. correcta* will reduce the number of odorant molecules diffusing into sensillum lumen and decrease the activation of olfactory receptors on neurons, which lead to the decreased EAG responses and olfactory deficit in LTC. These results suggest that we can control olfactory behaviors of the guava fruit fly by regulating nanopore numbers.

In summary, our results first reported abnormal antennal olfactory sensillum phenotypes in a long-term laboratory rearing colony of *B. correcta*, and further revealed that the reduction in nanopore numbers of olfactory sensilla impaired olfaction. These results will provide a platform to further study bulge and nanopore formation and nanopore-targeted pest control technologies of Tephritidae fruit flies.

## Figures and Tables

**Table 1:** Morphological characteristics of the antennal olfactory sensilla in the wild colony (WC) and long-term laboratory rearing colony (LTC) of *Bactrocera correcta*.

Olfactory Sensilla	WC				LTC				
	Length (μm)	Middle Width (μm)	Wall Nanopore	Nanopore Diameter (nm)	Length (μm)	Middle Width (μm)	Wall Bulge	Wall Nanopore	Nanopore Diameter (nm)
Grooved	3.23 ± 0.07 ^e^	0.50 ± 0.01 ^e^	–	–	3.26 ± 0.06 ^e^	0.50 ± 0.01 ^e^	–	–	–
Trichoid Ⅰ	20.41 ± 0.22 ^a^	1.19 ± 0.08 ^b^	+	22.8 ± 0.3 ^b^	20.70 ± 0.18 ^a^	1.19 ± 0.01 ^b^	+	+	22.6 ± 0.3 ^b^
Trichoid Ⅱ	12.06 ± 0.15 ^b^	1.01 ± 0.01 ^d^	+	23.3 ± 0.2 ^b^	12.09 ± 0.10 ^b^	1.01 ± 0.01 ^d^	+	+	23.3 ± 0.2 ^b^
Basiconica Ⅰ	10.80 ± 0.22 ^c^	1.31 ± 0.01 ^a^	+	43.7 ± 0.2 ^a^	10.87 ± 0.15 ^c^	1.31 ± 0.01 ^a^	+	+	43.9 ± 0.3 ^a^
Basiconica Ⅱ	7.72 ± 0.19 ^d^	1.09 ± 0.01 ^c^	+	43.5 ± 0.1 ^a^	7.87 ± 0.09 ^d^	1.09 ± 0.01 ^c^	+	+	43.7 ± 0.2 ^a^

Values represent means ± standard error, values followed by the different letters on the same column are significantly different (*p* < 0.05). The symbols “–” indicated that nanopores or bulges were not present. The symbols “+” indicated that nanopores or bulges were present. Twenty sensilla of each type of sensilla were measured.

## Data Availability

The authors confirm that the data supporting the findings of this study are available within the article.

## Supplementary Materials

The following supporting information can be downloaded at https://www.mdpi.com/article/10.3390/insects13060535/s1: Figure S1: Different collapse-like bulges and nanopores coexisted in the single basiconic sensillum in LTC. Table S1: The size of the antennal flagellum in WC and LTC groups. Table S2: The mean number of five morphological types of antennal olfactory sensilla in WC and LTC groups. Table S3: The mean nanopore number of four morphological types of olfactory sensilla in WC and LTC groups. Table S4: The EAG responses of WC and LTC groups to two odorants. Table S5: The total response rate and right response rate of WC and LTC groups to two odorants.

## References

[1] Wang X.J. (1996). The fruit flies (Diptera: Tephritidae) of the East Asian region. Acta Zool. Sin..

[2] Vargas R.I., Piñero J.C., Leblanc L. (2015). An overview of pest species of *Bactrocera* fruit flies (Diptera: Tephritidae) and the integration of biopesticides with other biological approaches for their management with a focus on the Pacific region. Insects.

[3] White I.M., Elson-Harris M.M. (1992). Fruit Flies of Economic Significance: Their Identification and Bionomics.

[4] Allwood A.J., Chinajariyawong A., Drew R.A.I., Hamacek E.L., Hancock D.L., Hengsawad C., Jinapin J., Jirasurat M., Krong K.C., Leong C.T.S. (1999). Host plant records for fruit flies (Diptera: Tephritidae) in South East Asia. Raffles Bull. Zool..

[5] United States Department of Agriculture Provisional List of Host Plants of Guava Fruit Fly, Bactrocera correcta (Bezzi) (Diptera: Tephritidae). https://www.aphis.usda.gov/plant_health/plant_pest_info/fruit_flies/downloads/host-lists//bactrocera-correcta-provisional-host-plants.pdf.

[6] Drew R.A.I., Raghu S. (2002). The fruit fly fauna (Diptera: Tephritidae: Dacinae) of the rainforest habitat of the Western Ghats, India. Raffles Bull. Zool..

[7] Liu X.F., Jin Y., Ye H. (2013). Recent spread and climatic ecological niche of the invasive guava fruit fly, *Bactrocera correcta*, in mainland China. J. Pest Sci..

[8] Shanbhag S.R., Müller B., Steinbrecht R.A. (1999). Atlas of olfactory organs of *Drosophila melanogaster*: 1. Types, external organization, innervation and distribution of olfactory sensilla. Int. J. Insect Morphol. Embryol..

[9] Yan X.Z., Deng C.P., Xie J.X., Wu L.J., Sun X.J., Hao C. (2017). Distribution patterns and morphology of sensilla on the antennae of *Plutella*
*xylostella* (L.)-A scanning and transmission electron microscopic study. Micron.

[10] Schneider D. (1969). Insect olfaction: Deciphering system for chemical messages. Science.

[11] Zacharuk R.Y. (1980). Ultrastructure and function of insect chemosensilla. Annu. Rev. Entomol..

[12] Ando T., Sekine S., Inagaki S., Misaki K., Badel L., Moriya H., Sami M.M., Itakura Y., Chihara T., Kazama H. (2019). Nanopore formation in the cuticle of an insect olfactory sensillum. Curr. Biol..

[13] Oh H.W., Jeong S.A., Kim J., Park K.C. (2019). Morphological and functional heterogeneity in olfactory perception between antennae and maxillary palps in the pumpkin fruit fly, *Bactrocera depressa*. Arch. Insect Biochem. Physiol..

[14] Steinbrecht R.A., Eguchi E., Tominaga Y. (1999). Olfactory receptors. Atlas of Arthropod Sensory Receptors-Dynamic Morphology in Relation to Function.

[15] Lee W.Y., Chang J.C., Hwang Y., Lin T.L. (1994). Morphology of the Antennal Sensilla of the Oriental Fruit Fly, *Dacus dorsalis* Hendel (Diptera: Tephrltidae). Zool. Stud..

[16] Hu F., Zhang G.N., Jia F.X., Dou W., Wang J.J. (2010). Morphological characterization and distribution of antennal sensilla of six fruit flies (Diptera: Tephritidae). Ann. Entomol. Soc. Am..

[17] Zheng W., Zhu C., Peng T., Zhang H. (2012). *Odorant receptor co-receptor Orco* is upregulated by methyl eugenol in male *Bactrocera dorsalis* (Diptera: Tephritidae). J. Insect Physiol..

[18] Liu H., Zhao X.F., Fu L., Han Y.Y., Chen J., Lu Y.Y. (2017). *BdorOBP2* plays an indispensable role in the perception of methyl eugenol by mature males of *Bactrocera dorsalis* (Hendel). Sci. Rep..

[19] Liu Z., Liang X.F., Xu L., Keesey I.W., Lei Z.R., Smagghe G., Wang J.J. (2020). An antennae-specific odorant-binding protein is involved in *Bactrocera dorsalis* olfaction. Front. Ecol. Evol..

[20] Wee S.L., Chinvinijkul S., Tan K.H., Nishida R. (2018). A new and highly effective male lure for the guava fruit fly *Bactrocera correcta*. J. Pest Sci..

[21] Tokushima I., Orankanok W., Tan K.H., Ono H., Nishida R. (2010). Accumulation of phenylpropanoid and sesquiterpenoid volatiles in male rectal pheromonal glands of the guava fruit fly, *Bactrocera correcta*. J. Chem. Ecol..

[22] De Rose F., Corda V., Solari P., Sacchetti P., Belcari A., Poddighe S., Kasture S., Solla P., Marrosu F., Liscia A. (2016). *Drosophila* mutant model of parkinson’s disease revealed an unexpected olfactory performance: Morphofunctional evidences. Parkinson’s Dis..

[23] Wang Z.W., Wen P., Qu Y.F., Dong S.H., Li J.J., Tan K., Nieh J.C. (2016). Bees eavesdrop upon informative and persistent signal compounds in alarm pheromones. Sci. Rep..

[24] Gu H.Y., O’Dowd D.K. (2006). Cholinergic synaptic transmission in adult *Drosophila* Kenyon cells in situ. J. Neurosci..

[25] Zhang X.G., Wei C.M., Miao J., Zhang X.J., Wei B., Dong W.X., Xiao C. (2019). Chemical compounds from female and male rectal pheromone glands of the guava fruit fly, *Bactrocera correcta*. Insects.

[26] Deng S.Z., Li X.Y., Wang Z.M., Wang J.B., Han D.Y., Fan J.H., Zhao Q., Liu H., Wang X.S. (2021). Assessment of 2-allyl-4,5-dimethoxyphenol safety and attractiveness to mature males of *Bactrocera dorsalis* (Hendel). Ecotoxicol. Environ. Saf..

[27] Ju Q., Guo X.Q., Li X., Jiang X.J., Jiang X.G., Ni W.L., Qu M.J. (2017). Plant volatiles increase sex pheromone attraction of *Holotrichia parallela* (Coleoptera: Scarabaeoidea). J. Chem. Ecol..

[28] Wigglesworth V.B. (1948). The insect cuticle. Biol. Rev..

[29] Keil T.A., Steiner C. (1991). Morphogenesis of the antenna of the male silkmoth. *Antheraea polyphemus*, Ⅲ. Development of olfactory sensilla and the properties of hair-forming cells. Tissue Cell..

[30] Ray K., Rodrigues V. (1995). Cellular events during development of the olfactory sense organs in *Drosophila melanogaster*. Dev. Biol..

[31] Jhaveri D., Sen A., Rodrigues V. (2000). Mechanisms underlying olfactory neuronal connectivity in *Drosophila*-the atonal lineage organizes the periphery while sensory neurons and glia pattern the olfactory lobe. Dev. Biol..

[32] Dorer D.R., Rudnick J.A., Moriyama E.N., Christensen A.C. (2003). A family of genes clustered at the Triplo-lethal locus of *Drosophila melanogaster* has an unusual evolutionary history and significant synteny with *Anopheles gambiae*. Genetics.

[33] Shah N., Dorer D.R., Moriyama E.N., Christensen A.C. (2012). Evolution of a large, conserved, and syntenic gene family in insects. G3-Genes Genom Genet..

[34] Smith C.R., Morandin C., Noureddine M., Pant S. (2018). Conserved roles of *Osiris* genes in insect development, polymorphism and protection. J. Evolution. Biol..

[35] Derby C.D., Kozma M.T., Senatore A., Schmidt M. (2016). Molecular mechanisms of reception and perireception in crustacean chemoreception: A comparative review. Chem. Senses.

