# Supplementary material for: Abnormal Antennal Olfactory Sensilla Phenotypes Involved in Olfactory Deficit in Bactrocera correcta (Diptera: Tephritidae)
Source: Insects. 2022 Jun 10;13(6):535. doi: 10.3390/insects13060535 (PMC9224687; doi:10.3390/insects13060535)
Supplement: Supplementary file 1 [file insects-13-00535-s001.zip › insects-1689313-supplementary.pdf]

insects MDPI

Supplementary Material

Abnormal antennal olfactory sensilla phenotypes involved in olfactory deficit in *Bactrocera correcta* (Diptera: Tephritidae)

Kai-Fei Guo[a,b,1], Xiao-Mei Peng[a,1], Jie-Yu Tu[a,1], Chan Jin[a], Wan-Rong Zhang[a], Xi-Zhu Chen[a], Yong-Jun Liu[c], Hong-Guang Zha[d], Wei Shi[a] and Jun Cao[a,*]

[a] Yunnan Key Laboratory of Plant Reproductive Adaptation and Evolutionary Ecology and Centre for Invasion Biology, Institute of Biodiversity, School of Ecology and Environmental Science, Yunnan University, Kunming, 650504, Yunnan, China; gkf@yxnu.edu.cn (K.-F.G.); pxm17863997736@163.com (X.-M.P.); luckytutu-tujieyu@163.com (J.-Y.T.); chanooos@163.com (C.J.); zhangwr91@163.com (W.-R.Z.); xzchen334@163.com (X.-Z.C.); shiwei@ynu.edu.cn (W.S.)
[b] School of Chemistry, Biology and Environment, Yuxi Normal University, Yuxi, Yunnan 653100, China
[c] School of Chemical Science and Technology, Yunnan University, Kunming, Yunnan 650504, China; yjliu@ynu.edu.cn (Y.-J.L.)
[d] College of Life and Environment Sciences, Huangshan University, Huangshan, Anhui 245041, China; zhahg@tech.hsu.edu.cn (H.-G.Z.)
[1] Authors contributed equally to the paper
* Correspondence author: J. Cao, juncao@vip.163.com

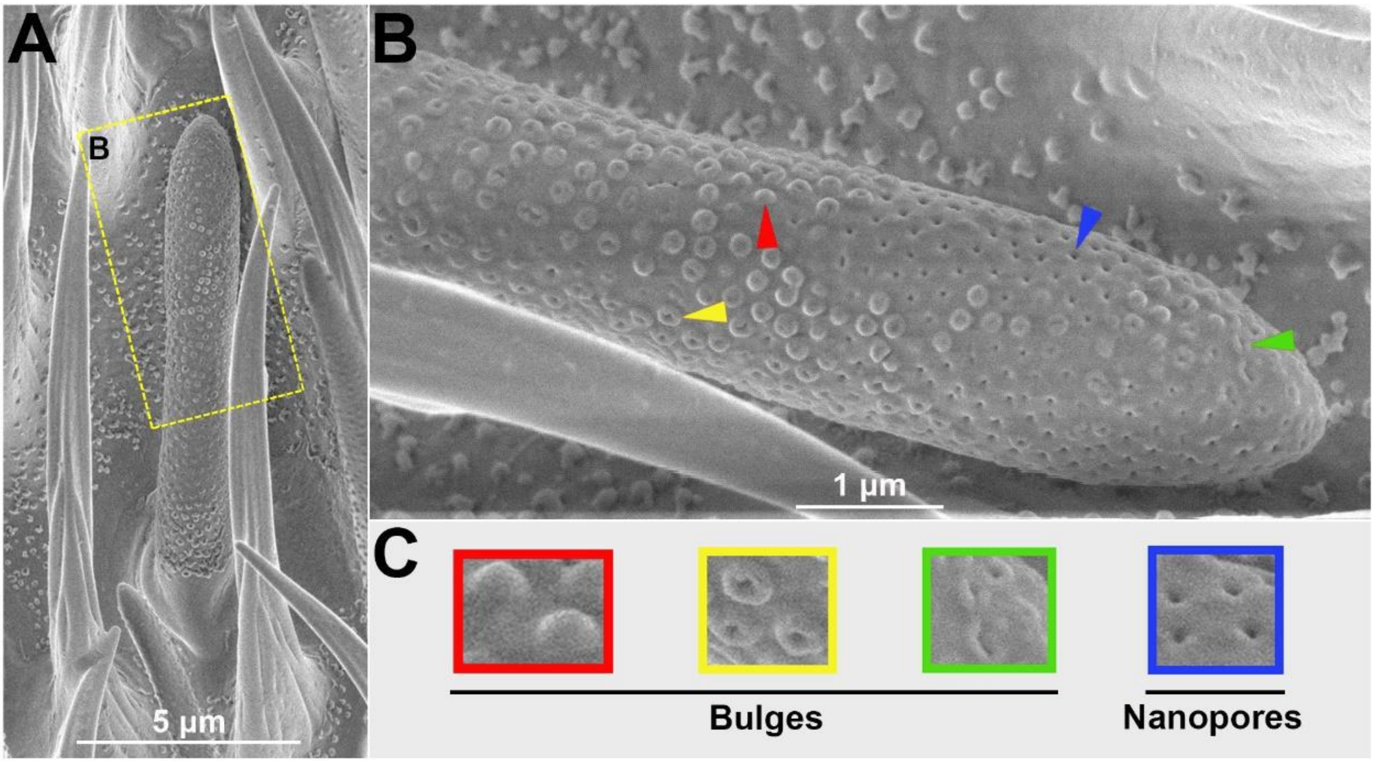

Figure S1. Different collapse-like bulges and nanopores coexisted in the single basiconic sensillum in LTC. (**A**) A SEM image of a basiconic sensillum. (**B**) Magnified image of a part (yellow dotted box) of the basiconic sensillum, showing full bulges (red arrowhead), slight collapse-like bulges (yellow arrowhead), heavy collapse-like bulges (green arrowhead) and pits (blue arrowhead). (**C**) Bulge and nanopore images extracted from (B).

Table S1. The size of the antennal flagellum in the WC and LTC groups.

Insects	Antennal flagellum	
	Length (μm)	Width (μm)

WC	571.9 ± 3.5 [a]	170.6 ± 2.3 [a]
LTC	566.9 ± 2.5 [a]	169.9 ± 2.6 [a]

Values represent means ± standard error, values followed by the same letter on the same column are not significantly different ($p > 0.05$). Sixteen antennae from each group were measured.

Table S2. The mean number of five morphological types of antennal olfactory sensilla in the WC and LTC groups.

Sensilla types	Number of sensilla	
	WC	LTC
Trichoid I	1284.7 ± 28.0 [a]	1294.3 ± 25.2 [a]
Trichoid II	372.5 ± 5.9 [c]	392.7 ± 18.8 [c]
Basiconica I	227.8 ± 8.9 [e]	245.2 ± 20.4 [e]
Basiconica II	423.2 ± 31.6 [b]	472.8 ± 28.1 [b]
Grooved	318.8 ± 12.8 [d]	327.3 ± 19.4 [d]

Values represent means ± standard error, values followed by the different letters on the same column are significantly different ($p < 0.05$). Six antennae from each group were examined.

Table S3. The mean nanopore number of four morphological types of olfactory sensilla in the WC and LTC groups.

Sensilla type	Number of nanopores	
	WC	LTC
Trichoid I	234.6 ± 5.1 [b]	118.3 ± 18.3 [b]
Trichoid II	136.6 ± 2.7 [d]	74.5 ± 11.6 [c]
Basiconica I	426.4 ± 4.4 [a]	176.8 ± 31.7 [a]
Basiconica II	194.4 ± 4.1 [c]	112.7 ± 16.9 [b]

Values represent means ± standard error, values followed by the different letters on the same column are significantly different ($p < 0.05$). Twenty sensilla from each type of sensilla were examined.

Table S4. The EAG responses of the WC and LTC groups to two odorants.

Insects	EAG responses (mV)	
	Methyl eugenol	β-caryophyllene
WC	1.46 ± 0.13 [a]	1.35 ± 0.11 [a]
LTC	0.53 ± 0.05 [b]	0.42 ± 0.05 [b]

Values represent means ± standard error, values followed by the different letters on the same column are significantly different ($p < 0.001$). Fifteen insects from WC or LTC were respectively used for EAG recording to each odorant.

Table S5. The total response rate and right response rate of the WC and LTC groups to two odorants.

Insects	Total response rate (%)		Right response rate (%)	
	Methyl eugenol	β-caryophyllene	Methyl eugenol	β-caryophyllene
WC	90.8 ± 3.0 [a]	93.3 ± 2.5 [a]	83.3 ± 2.1 [a]	80.8 ± 2.4 [a]
LTC	55.0 ± 3.7 [b]	60.0 ± 2.9 [b]	43.3 ± 2.1 [b]	48.3 ± 2.1 [b]

Values represent means ± standard error, values followed by the different letters on the same column are significantly different ($p < 0.001$). Six replications from WC or LTC were respectively used for behavioral bioassay to each odorant.
